# Assessment of an Anticancer Effect of the Simultaneous Administration of MM-129 and Indoximod in the Colorectal Cancer Model

**DOI:** 10.3390/cancers16010122

**Published:** 2023-12-26

**Authors:** Iwona Kwiatkowska, Justyna Magdalena Hermanowicz, Robert Czarnomysy, Arkadiusz Surażyński, Krystyna Kowalczuk, Joanna Kałafut, Alicja Przybyszewska-Podstawka, Krzysztof Bielawski, Adolfo Rivero-Müller, Mariusz Mojzych, Dariusz Pawlak

**Affiliations:** 1 Department of Pharmacodynamics, Medical University of Bialystok, Mickiewicza 2C, 15-222 Bialystok, Poland; justyna.hermanowicz@umb.edu.pl (J.M.H.); dariusz.pawlak@umb.edu.pl (D.P.); 2 Department of Clinical Pharmacy, Medical University of Bialystok, Mickiewicza 2C, 15-222 Bialystok, Poland; 3 Department of Synthesis and Technology of Drugs, Medical University of Bialystok, Mickiewicza 2C, 15-222 Bialystok, Poland; robert.czarnomysy@umb.edu.pl (R.C.); krzysztof.bielawski@umb.edu.pl (K.B.); 4 Department of Medicinal Chemistry, Medical University of Bialystok, Mickiewicza 2C, 15-222 Bialystok, Poland; arkadiusz.surazynski@umb.edu.pl; 5 Department of Integrated Medical Care, Medical University of Bialystok, ul. M Skłodowskiej-Curie 7A, 15-096 Bialystok, Poland; krystyna.kowalczuk@umb.edu.pl; 6 Department of Biochemistry and Molecular Biology, Medical University of Lublin, Chodźki 1, 20-093 Lublin, Poland; joanna.kalafut@umlub.pl (J.K.); alicja.przybyszewska-podstawka@umlub.pl (A.P.-P.); adolfo.rivero-muller@umlub.pl (A.R.-M.); 7 Faculty of Health Science, Collegium Medicum, The Mazovian Academy in Plock, Plac Dabrowskiego 2, 09-402 Plock, Poland; mmojzych@yahoo.com

**Keywords:** MM-129, indoximod, colon cancer, kynurenine pathway, immunooncology, zebrafish

## Abstract

**Simple Summary:**

The discovery of the involvement of the kynurenine pathway in carcinogenesis has prompted significant changes in pharmacotherapy strategies. One such approach involves the simultaneous targeting of an ongoing process in cancer cells and inhibiting the abovementioned pathway. Based on this approach, we investigated the anticancer effect of combining a 1,2,4-triazine derivative, MM-129, together with an inhibitor of the kynurenine pathway, indoximod, in a colon cancer model. The obtained results support the efficacy of this strategy, providing a basis for future in-depth analyses.

**Abstract:**

(1) Background: The purpose of the given study was to examine the antitumor activity of the simultaneous administration of MM-129, a 1,2,4-triazine derivative, and indoximod (IND), the kynurenine pathway inhibitor, toward colon cancer. (2) Methods: The efficiency of the co-administration of the studied compounds was assessed in xenografted zebrafish embryos. Then, the effects of the combined administration of compounds on cellular processes such as cell viability, apoptosis, and intracellular signaling pathways were evaluated. In vitro studies were performed using two colorectal cancer cell lines, namely, DLD-1 and HT-29. (3) Results: The results indicated that the simultaneous application of MM-129 and indoximod induced a stronger inhibition of tumor growth in zebrafish xenografts. The combination of these compounds intensified the process of apoptosis by lowering the mitochondrial potential, enhancing the externalization of phosphatidylserine (PS) and activation of caspases. Additionally, the expression of protein kinase B (AKT) and indoleamine 2,3-dioxygenase-(1IDO1) was disrupted under the applied compound combination. (4) Conclusions: Simultaneous targeting of ongoing cell signaling that promotes tumor progression, along with inhibition of the kynurenine pathway enzyme IDO1, results in the enhancement of the antitumor effect of the tested compounds against the colon cancer cells.

## 1. Introduction

The kynurenine pathway (KP) is one of the tryptophan (TRP) metabolism pathways. Elements of the path include rate-limiting enzymes, such as IDO1, IDO2, and TDO2 (indoleamine 2,3-dioxygenase-1, indoleamine 2,3-dioxygenase-2, and tryptophan 2,3-dioxygenase), and arising metabolites, i.e., kynurenine and kynurenic acid. Mounting evidence indicates a major role of KP in oncogenesis via a specific process called immunoediting. This phenomenon assumes that tumor cells disrupt the microenvironment to promote immune evasion. Elements of KP are highly involved in this unfavorable process. For example, overexpression of IDO1, IDO2, and TDO2 results in drug resistance, enhanced metastasis formation, intense cancer cell proliferation, impeded apoptosis, and a poor prognosis for patients [1,2,3,4,5]. To explain the role of these enzymes in the development of immunosuppressive microenvironment, it should be mentioned that their overexpression and high enzymatic activity on cancer cells lead to a local depletion of TRP. This effect was previously linked to the impairment of T cells, dendritic cells, mastocytes, and macrophage function with concomitant intense tumor growth [6,7]. There is also some evidence of a precancerous effect of KP elements directly exerted on tumor cells. Indeed, IDO1 expression correlates with the enhanced aggressiveness of cancer cells via the dysregulation of the markers linked with epithelial-to-mesenchymal transition such as E-cadherin, N-cadherin, and vimentin [8,9]. Additionally, some researchers noticed an enhancement of angiogenesis in the presence of IDO1 [10]. All this has fueled the search for molecules that inhibit the activity of the KP pathway as an innovative approach to cancer treatment.

One such molecule is indoximod (IND). It exerts pleiotropic effects on immune regulation by reversing an inhibition of mTOR in T cells and restricting the activity of GCN2 kinase in effector T cells. That restores their proliferation and anticancer capability [11]. Via the modulation of aryl hydrocarbon receptor (AhR) activity on T cells, it promotes their differentiation into the Th subpopulation and diminishes their polarization into immunosuppressive Treg cells. It also leads to the upregulation of the transcription of RORC along with the downregulation of the transcription of FOXP3 factors [12]. In dendritic cells, IND, via AhR interaction, leads to a downregulation of IDO expression, which improves dendritic cells’ ability for antigen presentation and antitumor response. Moreover, IND intensifies the efficacy of DNA-damaging chemotherapy [13] and exerts a stronger antitumor effect in combination with anti-PD-1/PD-L1 antibodies (programmed cell death protein-1/programmed death ligand-1) [14]. So far, research on the IND mechanism of action and effectiveness has focused mainly on its immunological effects. Reports on its impact on processes ongoing directly in cancer cells are limited.

In our previous study, we developed a small 1,2,4-triazine derivative (MM-129) and proved its anticancer activity in vitro in zebrafish and mouse xenografts [15]. We showed, that MM-129 exerts its favorable effect on colon cancer cells by inhibiting their crucial signaling pathways, such as PI3K/AKT/mTOR and Bruton’s tyrosine kinase (BTK), together with decreasing PD-L1 expression [16]. Moreover, MM-129 enhances caspases activity with concomitant increased apoptosis of colon cancer cells. Additionally, we proved a favorable safety profile of the molecule [17]. 

Since it is known that IND enhances the effectiveness of compounds affecting the abovementioned pathways, and MM-129 combines the features of classic chemotherapeutics by blocking the immune checkpoint, we hypothesized that the co-administration of these compounds may exert a stronger antitumor effect. As a continuation of our previous research, we designed and conducted a study that aimed to verify this idea and to assess an antitumor activity of combined administration of MM-129 and IND in an experimental colon cancer model. In order to evaluate this, we established zebrafish xenografts using two lines of colorectal cancer (DLD-1 and HT-29). We also conducted mechanistic studies using zebrafish embryos and colon cancer cells (DLD-1 and HT-29) to evaluate the intracellular signaling pathways and other molecular events that might be involved in the antitumor effect exerted by the compound combination. 

## 2. Materials and Methods

### 2.1. Zebrafish Drug Screening Assay

EU Directive 2010/63/EU of 22 September 2010 regulates the criteria for the inclusion of animals in scientific research. According to this document, zebrafish (*Danio rerio*) embryo and eleutheroembryo cultures, meaning their earliest life stages, are regarded as equivalent to in vitro cell culture. For this reason, they are not subject to the regulatory frameworks dealing with animal experiments. Nevertheless, experiments with free-feeding larvae older than 120 hpf (hours post-fertilization) of development are classified as animal experiments. This is why they require adequate permissions. The experiments planned in our study were conducted only on zebrafish larvae younger than 120 hpf; therefore, ethics approval was not required. The conditions provided to the zebrafish were as follows: 28.5 °C in E3 buffer in 30 L aquaria. That made a rate of one fish per liter of water. Light/darkness cycles were, respectively, 14/10 h. The zebrafish were fed in accordance with the guidelines established by the Research Animals Department of the Royal Society for the Prevention of Cruelty to Animals (RSPCA). 

### 2.2. Zebrafish Xenograft Injection

Colon cancer DLD-1 and HT-29 cell lines were labeled with Vybrant Dil (Invitrogen, Waltham, MA, USA) according to the manufacturer’s protocol. Stained cells were re-suspended in Dulbecco’s modified Eagle’s medium (DMEM) at the final concentration of 1 × 10^7^ cells/mL. Zebrafish larvae were manually dechorionated 24 hpf and injected with cells 48 hpf. Cancer cells were loaded into a glass needle pulled by a P-1000 Next Generation Micropipette Puller (Sutter Instrument Company, Novato, CA, USA). Approximately 300 labeled cells were injected into the interior yolk space of each larva using an electronically regulated air-pressure microinjector (Narishige IM-300 Microinjector, Tokyo, Japan). After injection, the zebrafish larvae with transplanted cells (DLD-1 *n* = 80 and HT-29 *n* = 80) were randomly assigned to control and drug treatment groups. DLD-1 and HT-29 xenografts (72 hpf) were incubated with MM-129 (10 µM), IND (200 µM), or compound combination (MM-129 + IND) for 48 h. MM-129 was dissolved in dimethyl sulfoxide (DMSO), below 0.1%, and IND was dissolved in water. The control group was incubated in E3 buffer enriched with DMSO in the concentration 0.1%. The zebrafish xenografts were cultured in an E3 buffer at 34 °C until the end of the experiment (120 hpf). 

### 2.3. In Vivo Imaging

The zebrafish larvae were anesthetized by 0.04 mg/mL ethyl 3-aminobenzoate methanesulfonate tricaine before imaging. Images of xenografts were acquired in 48 hpf and in 120 hpf, using an EVOS M5000 Imaging System with the filter Cy5 (excitation: 628 nm; emission: 692 nm). 

### 2.4. RNA Extraction and Quantitative Analysis

Total ribonucleic acid (RNA) from 20 fish with xenografts in each studied group was isolated using an ExtractMe Total RNA kit (Blirt, Gdansk, Poland) according to the manufacturer’s protocol. The isolated RNA was used for cDNA synthesis through a High-Capacity cDNA Reverse Transcription Kit with the addition of an RNase Inhibitor (Applied Biosystems, Waltham, MA, USA). Zebrafish *gpdh* (forward 5′-GTGGAGTCTACTGGTGTCTTC-3′, reverse 5′-GTGCAGGAGGCATTGCTTACA-3′) was used as a housekeeping gene. The human *GAPDH* gene (forward 5′-CTCTGCTCCTCCTGTTCGAC-3′, reverse 5′-GCCCAATACGACCAAATCC-3′) was used to quantify the number of human cells in the xenografted zebrafish larvae. All primers used for qPCR were tested for specificity and sensitivity. Quantitative real-time polymerase chain reaction (qPCR) expression analysis was then performed with the LightCycler^®^ 480 II instrument (Roche, Basel, Switzerland) in triplicate on 96-well plates using PowerUp SYBR Green Master Mix (Applied Biosystems). Relative mRNA expression was calculated using the delta CT subtraction and normalized [18,19].

### 2.5. Zebrafish Egg Proliferation Assay

Zebrafish embryos were obtained from mating adults, maintained and raised as described previously [15]. Zygote period cleaving eggs were transferred to six-well plastic cell culture plates filled with embryo medium E3. The eggs (20 per well) were exposed to MM-129 (10 µM), IND (200 µM), or compound combination (MM-129 + IND) for 3 h. The final volume of the medium in each well was 2 mL. DMSO was used as a MM-129 solvent, and water was used as an IND solvent. The final concentration of DMSO in the wells did not exceed the concentration above 0.1%, which means that it was within the range ensuring no harmful effects of the solvent. Embryos from the control group were incubated in an E3 medium in the presence of 0.1% DMSO. During the experiment, all embryos were observed and the moment when characteristic changes occurred in all embryos of a given group was selected as the point of triggering the effect of the test compound. Each test was independently repeated three times. Cell division and zebrafish eggs development were conducted with the use of SteREO Discovery. A V8 stereo microscope (Zeiss, Jena, Germany) was used. Photos of ongoing processes were taken once every 15 min within the first three hours of incubation.

### 2.6. Cell Culture

Two human colorectal adenocarcinoma cell lines DLD-1 (CCL-221) and HT-29 (HTB-38) were purchased from the American Type Culture Collection (ATCC, Manassas, VA, USA). They were chosen based on their features. The use of the DLD-1 line allows for the checking of the sensitivity of cells histologically the most similar to a primary tumor to the tested compounds. On the other hand, the inclusion of cells from the HT-29 line allows us to assess whether multidrug resistance, absorption of nutrients, and chemically induced differentiation of enterocytes emerge. DLD-1 cells (passage number range of 6–8) were cultured in RPMI 1640 medium (Sigma, St. Louis, MO, USA) and HT-29 cells in McCoy’s 5a medium (ATCC). Both media were complemented with 10% of fetal bovine serum (FBS) and 1% antibiotics: penicillin/streptomycin. The cells were cultured in 100 mm plates (Sarstedt, Newton, NC, USA) and stored in the incubator. The conditions maintained in the incubator ensured optimal cell growth and were 5% CO_2_, 37 °C, and humidity in a range of 90–95%. After obtaining 70–80% confluency (a subconfluent cell culture), the cells were detached with 0.05% trypsin and 0.02% EDTA phosphate-buffered saline without calcium and magnesium (Corning, Corning, NY, USA). The cells were treated with compound solutions that were prepared as follows: MM-129 was dissolved in DMSO, below 0.1%, and IND was dissolved in water.

### 2.7. Cell Viability Assay

Cytotoxicity of the tested compound combination was evaluated by 3-(4,5-dimethylthiazol-2-yl)-2,5-diphenyltetrazolium bromide (MTT) assay. DLD-1 and HT-29 cells were seeded in six-well plates “Nunc” at a density of 5 × 10^5^ cells/well and incubated for 24 h in optimal growth conditions. Next, MM-129 (10 µM), IND (200 µM), or a compound combination (MM-129 + IND), were added in duplicate, and the plates were incubated for another 24 h. After the incubation, the plates were washed with PBS three times. Then, 1 mL of PBS and 50 µL of MTT solution were added, and the incubation was continued for 15 min. MTT solution was prepared as follows: 5 mg of MTT was dissolved in 1 mL of PBS. MTT tetrazolium is converted in viable cells into purple crystals of formazan, which does not appear in dead cells. Next, the supernatant was removed, and formazan crystals were dissolved in DMSO. In the following step, Sorensen’s buffer was added. After that the absorbance was measured at a wavelength of 570 nm. The absorbance result obtained in the control was taken as 100%, and the viability of the cells incubated with the tested compounds was shown as a percentage of the control cells. The values from the samples were obtained from three independent experiments performed in duplicate (*n* = 6).

### 2.8. Flow Cytometry Assessment of Annexin V Binding

Induction of apoptosis was examined by flow cytometry, with the use of an Apoptosis Detection Kit II. The test principle is based on the observation that during programmed cell death (PCD), the phosphatidylserine (PS) is transferred to the cell surface from the inner side of the cells. DLD-1 and HT-29 cells (70–80% of confluence) were incubated (24 h) with MM-129 (10 µM), IND (200 µM), or a compound combination (MM-129 + IND). After 24 h incubation with the given compounds, the cells were dyed with annexin V-FITC and propidium iodide (PI). Double staining allows for the identification of viable, early, late apoptotic, and necrotic cells. Cells in which apoptosis was induced by the addition of 2 µL of 3% formaldehyde were used as a positive control. The cells were placed in a refrigerator for 15 min to induce apoptosis. Three controls were made: the first contained control cells and propidium iodide; the second, control cells and annexin V-FITC; and the third, control cells and propidium iodide and annexin V-FITC. To establish a negative control, cells cultured in a medium without the tested compounds were used. The test was independently repeated three times. The experiment was performed using the BD FACSCanto II flow cytometer, and the results were parsed with FACSDiva software (version 6.1.3, BD Biosciences Systems, San Jose, CA, USA). 

### 2.9. Analysis of Mitochondrial Membrane Potential 

Flow cytometry was conducted to test occurring changes in the mitochondrial membrane potential (MMP). For this purpose, the JC-1 MitoScreen kit (BD Biosciences, San Jose, CA, USA) was used. The test principle is that in normal cells, lipophilic dye JC-1 (1,10,3,30-tetraethyl- 5,50,6,60-tetrachloroimidacarbocyanine iodide), which aggregates in the mitochondrial matrix. In turn, in apoptotic and necrotic cells, this dye diffuses out of mitochondria, which is manifested by the green fluorescent cell staining. DLD-1 and HT-29 colon cancer cell lines covering about 80% of the plate were incubated with MM-129 (10 µM), IND (200 µM), or compound combination (MM-129 + IND) for 24 h in an incubator, with the same conditions as described above, i.e., 37 °C and 5% CO_2_. After the incubation time, the medium was removed. The cells were washed two times with the required buffer. Subsequently, the cells were suspended in a 10 µg/mL JC-1 dye and incubated in the dark for 15 min. Before the analysis, the cells were washed with PBS and then analyzed using a BD FacsCanto II flow cytometer. The results were assessed using BD FACSDiva software (version 6.1.3, BD Biosciences Systems, San Jose, CA, USA). The test was independently repeated three times.

### 2.10. Caspase Activity Assays

Caspase-8, -10, and -3/7 activity were assessed with the adequate kits (caspase-8: FLICA Caspase-8 Assay Kit, caspase-10: FLICA Caspase-10 Assay Kit, caspase-3/7: FLICA Caspase-3/7 Assay Kit, Mont-Royal, QC, Canada). DLD-1 and HT-29 (70–80% of confluence) were incubated with MM-129 (10 µM), IND (200 µM) or compound combination (MM-129 + IND) for 24 h. After the incubation time, the cells were washed twice with cold PBS. Next, the cells were resuspended in the required buffer (93 µL was gently mixed with 5 µL required FLICA and 2 µL Hoechst 33342) and incubated at 37 °C for 60 min. Afterward, the cells were washed twice with apoptosis wash buffer and centrifuged at 300× *g*. Cells from samples prepared in this manner were resuspended in 100 µL buffer and labeled with 10 µg/mL propidium iodide. The experiment was performed using the BD FACSCanto II flow cytometer. The results were analyzed with FACSDiva software (version 6.1.3, BD Biosciences Systems, San Jose, CA, USA). The values were obtained from three independent experiments performed in duplicate (*n* = 6).

### 2.11. Capillary Protein Separation and Immunodetection

DLD-1 and HT-29 cells were incubated for 24 h with MM-129 (10 µM), IND (200 µM), or compound combination (MM-129 + IND). After the incubation, cells were collected and pellets were lysed in 100 µL RIPA buffer enriched with protease and phosphatase inhibitors (Sigma-Aldrich). Cellular membranes were disrupted by sonification. The level of total protein concentration was determined using the bicinchoninic acid (BCA) method according to the manufacturer’s protocol (Thermo Fisher Scientific, Waltham, MA, USA). Samples were brought to equal amounts of protein (0.4 mg/mL) and were loaded into a cartridge. Protein samples were separated by capillary electrophoresis using the 12–230 kDa Jess Separation Module (ProteinSimple, San Jose, CA, USA) following the manufacturer’s instructions. Target proteins were detected with the following monoclonal antibodies: mouse anti-IDO1 (Sigma-Aldrich, Cat# SAB3701446, 1:100), mouse anti-AKT (Sigma-Aldrich, Cat# 05-591, 1:100), and mouse anti-β-actin (Sigma Aldrich, Cat #A2228, 1:100). For detection, the anti-mouse module for the Jess (DM-002, ProteinSimple) kit was used, which includes luminol-S, peroxide, antibody diluent 2, streptavidin-HRP, and anti-mouse secondary antibody. Results are presented as blot images generated by the Compass software (Compass for SW software v5.0.1), based on chemiluminescence signal for each targeted protein. To confirm loaded protein level and to verify normalized protein amount, detection of housekeeping protein β-actin was performed. For statistical analysis the chemiluminescence of secondary antibody signal peaks were chosen. The values were obtained from three independent experiments done in duplicate (*n* = 6).

### 2.12. Statistical Analysis

Shapiro–Wilk’s test of normality was used for data distribution analysis. The normally distributed data were expressed as mean ± standard deviation (SD). Multiple group comparisons were performed by one-way analysis of variance (ANOVA), and significant differences between the groups were assessed using the Tukey–Kramer test and column statistics. Calculations were performed using GraphPad 6 Prism software (La Jolla, CA, USA). The differences were considered statistically significant when * *p* < 0.05.

## 3. Results

### 3.1. Compound Combination Had a Favorable Impact on Tumor Growth in Zebrafish Xenografts

**Figure 1:**
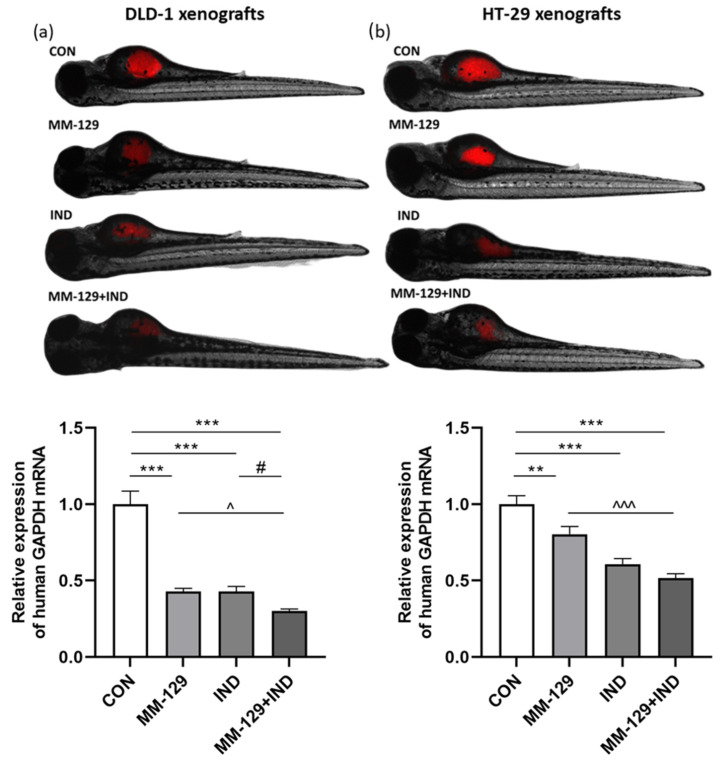
Impact of MM-129 (10 µM), IND (200 µM) or a combination of these agents (MM-129 + IND) on tumor development in DLD-1 (**a**), and HT-29 (**b**) zebrafish xenografts, and the expression of human *GAPDH* mRNA was determined at 120 hpf. Data were presented as mean ± standard deviation (SD) and analyzed using one-way analysis of variance (ANOVA). *n* = 20 each group; ** *p* < 0.01, *** *p* < 0.001 vs. CON; ^ *p* < 0.05, ^^^ *p* < 0.001 vs. MM-129; # *p* < 0.05 vs. IND.

The antitumor activity of the compound combination (MM-129 + IND) was assessed in the zebrafish model of xenotransplantation. DLD-1 and HT-29 cells, stained with Vybrant Dil, were implemented into the yolk sac of 48 hpf zebrafish embryo (*n* = 80 each group). Established xenografts (72 hpf) were assigned to four groups: control, exposed to MM-129 (10 µM), exposed to IND (200 µM), or exposed to MM-129 + IND for 48 h. After the allotted time, the larvae were collected and RNA was extracted to quantify the presence of human cells vs. fish tissue by measuring the human *GAPDH* gene vs. zebrafish gadph gene via qRT-PCR. In both DLD-1 and HT-29 xenografts, 48 h exposition to MM-129 or IND alone resulted in a statistically significant reduction of the human *GAPDH* presence. In both groups of DLD-1 cells exposed to a single compound, we obtained a reduction in the presence of the human *GAPDH* gene at a comparable level. On the other hand, in HT-29 xenografts, IND alone resulted in a more pronounced decrease in *GAPDH* gene presence than individuals exposed to MM-129 alone. What is valuable is that the decrease in the investigated gene presence was strengthened after the co-administration of compounds; however, this was more noticeable in DLD-1 xenografts. This led us to conclude that the inhibition of tumor development was most evident in the MM-129 + IND group, and that even though both tested compounds bear antitumor potential, this property was enhanced by their co-administration (Figure 1).

### 3.2. Antiproliferative Activity of Compound Combination in a Zebrafish Model

Zebrafish as a model organism in oncological research is also used to assess antiproliferative properties, whereas cell division is analyzed during embryogenesis. Briefly, in the first 45 min, zebrafish eggs form the stage of two cells, later in every 15 min the number of cells duplicate, creating the stages of 4, 8, and 64 cells within 1, 1.25, and 2 hpf, respectively. The fact that the cell division is easy to observe and document as microscopic photos makes it a widely used platform for evaluating the antiproliferative activity of compounds of interest. 

**Figure 2:**
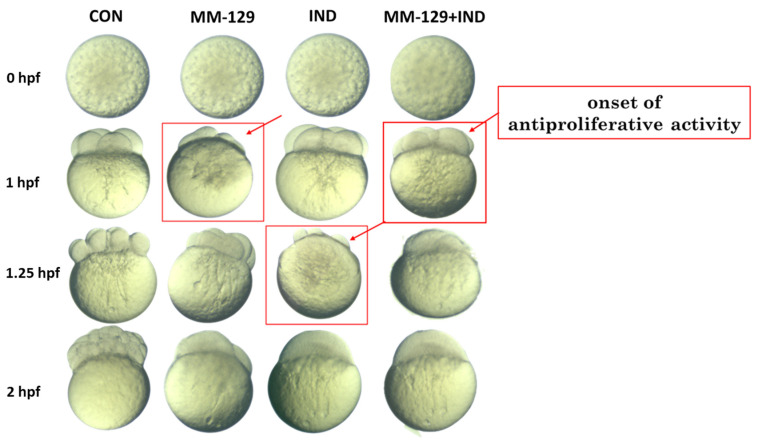
Effect of MM-129 (10 µM), IND (200 µM), and compound combination (MM-129 + IND) on cell division in the zebrafish embryo. Zebrafish eggs after 0, 1, 1.25, and 2 h of exposure to tested compounds; *n* = 20, hpf: hours post-fertilization.

In our experiment, we noticed a disruption in cell division in all experimental groups, while no disturbances were observed in the control group (Figure 2). However, the mentioned disturbances did not manifest at the same time spot. Only in the groups exposed to MM-129 and MM-129 + IND did we observe cell division arrest after 1 h. Cell division was also impaired by IND administrated alone; nevertheless, this effect occurred in the next stage of proliferation. The first changes in the development impairment were visible after 1.25 hpf, meaning on the stage of eight cells. After 2 h, we observed definitive proliferation inhibition in all experimental groups, while the development of untreated embryos was preserved without prejudice. This demonstrated the antiproliferative potential of both tested compounds; however, it suggested that MM-129 was more potent to inhibit cell division than IND alone. 

### 3.3. Antiproliferative Activity of Compound Combination in Colon Cancer Cells

**Figure 3:**
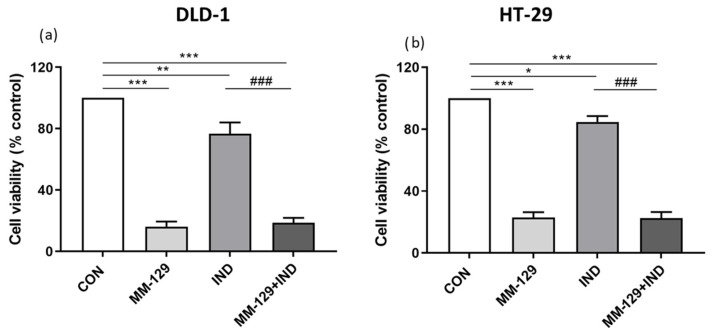
Viability of DLD-1 (**a**) and HT-29 (**b**) colon cancer cells treated for 24 h with MM-129 (10 µM), IND (200 µM), and compound combination (MM-129 + IND). Results are presented as mean values ± standard deviation (SD) from three independent experiments performed in duplicate. * *p* < 0.05, ** *p* < 0.01, *** *p* < 0.001 vs. CON; ### *p* < 0.001 vs. IND.

To verify observations made in zebrafish embryos, we conducted a classical MTT assay on DLD-1 and HT-29 cells. This allowed us to examine the effect of 24 h incubation with the tested compounds on the viability of cancer cells. In the course of the study, we found out that both compounds bore antiproliferative potential; however, when compared to the control, this effect was stronger in the group exposed to MM-129 than in the group exposed to IND alone. These results reflected observations made in zebrafish embryos. In the case of co-administration of MM-129 with IND, the obtained results were convergent for both tested cell lines and indicated a strong inhibition of cell viability. However, in the in vitro condition, the addition of IND did not increase the antiproliferative potential of MM-129 (Figure 3). The reason may be both the strong antiproliferative effect of MM-129 and the weak effect of IND in vitro, which requires the presence of immune cells to be fully effective.

### 3.4. Compound Combination Enhanced Apoptosis via Decreasing Mitochondrial Membrane Potential and Phosphatidylserine Externalization

These results made us take further steps to test whether the compound combination exerts a significant impact on the molecular processes driving cancer development. For this purpose, we conducted several experiments to investigate changes in cytotoxicity. One of the well-known hallmarks of programmed cell death is a loss of mitochondrial membrane potential (MMP). As a consequence, the release of apoptogenic factors and loss of oxidative phosphorylation might be detected.

**Figure 4:**
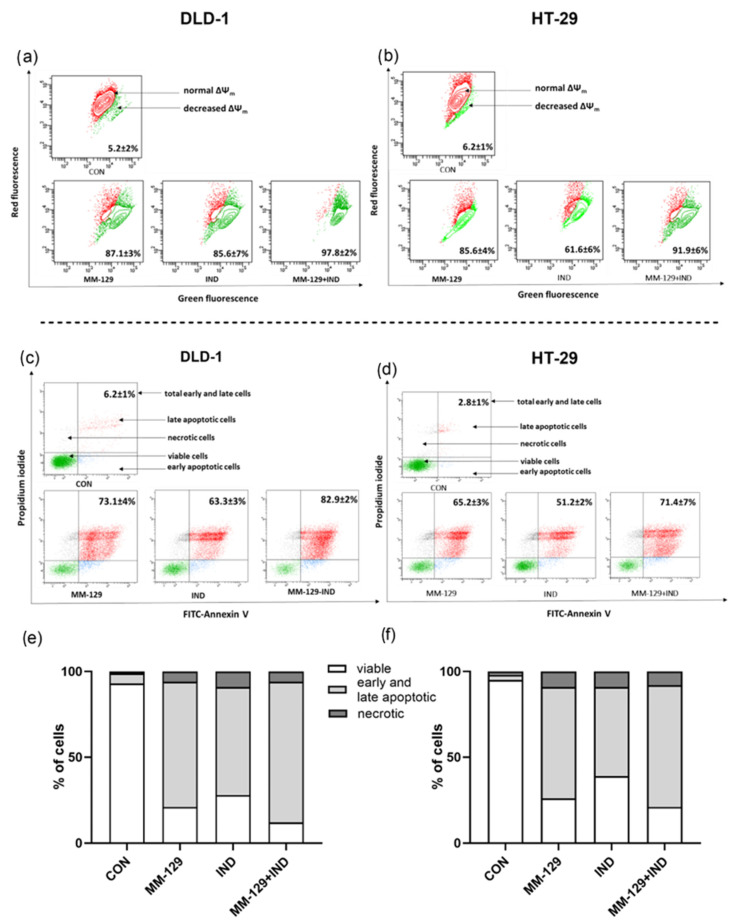
Representative dot-plots illustrating the loss of mitochondrial membrane potential, ΔΨm. (**a**,**b**) Flow cytometry dot-plots for the annexin V-FITC-propidium iodide assay (**c**,**d**) and quantitative chart illustrating the distribution of live, early, and late apoptotic and necrotic cells (**e**,**f**) of DLD-1 (**a**,**c**,**e**) and HT-29 (**b**,**d**,**f**) after 24 h exposition to MM-129 (10 µM), IND (200 µM), and MM-129 + IND. Values were obtained from three independent experiments.

Using flow cytometry, we investigated the proapoptotic effect exerted in vitro after 24 h exposition on the proposed compound combination. In DLD-1, MM-129 and IND administrated alone caused a decrease in MMP in 87.1 ± 3% and 85.6 ± 7% of cells, respectively. The addition of IND to MM-129 resulted in a decrease in MMP in 97.8 ± 2% (^ *p* < 0.05 vs. MM-129; # *p* < 0.05 vs. IND). In the HT-29 line, MM-129 caused a stronger reduction of MMP than IND (85.6 ± 4% vs. 61.6 ± 6% respectively; && *p* < 0.01). In the MM-129 + IND group, we observed a higher percentage of cells with decreased MMP compared to the IND group (91.9 ± 6% vs. 61.6 ± 6% respectively, ### *p* < 0.001) (Figure 4a,b).

To describe in detail the changes in the apoptosis process under the influence of the compound combination, we investigated its impact on the externalization of phosphatidylserine. Briefly, the translocation of phosphatidylserine from the internal surface to the external surface of the plasma membrane is one of the recognized mechanisms that allows for the recognition and elimination of apoptotic cells [20]. Phosphatidylserine externalization might be assessed with the use of flow cytometry and the double staining method, using annexin V and propidium iodide (PI). It is known that early apoptotic cells bind only with annexin V, late-apoptotic cells bind with both of the dyes, necrotic cells bind only with PI, and the living population does not bind with any of the dyes. Due to the selective binding of dyes by cells in different states, the test allowed us to characterize the occurring cell death in detail and enabled us to conduct a more in-depth assessment of the impact of the tested approach on apoptosis.

In both cell lines exposed to a single compound, apoptosis (early and late) was detected in a higher percentage of cells incubated for 24 h with MM-129 than in those incubated with IND (respectively, 73.1 ± 4% vs. 63.3 ± 3% in DLD-1; & *p* < 0.05; 65.2 ± 3% vs. 51.2 ± 2% in HT-29; & *p* < 0.05). In DLD-1, we observed an increase in apoptotic cells after the combined administration compared to MM-129 (^ *p* < 0.05) and IND (### *p* < 0.001) alone (Figure 4c–f).

### 3.5. The Addition of IND Intensified Caspase Activation Exerted by MM-129

**Figure 5:**
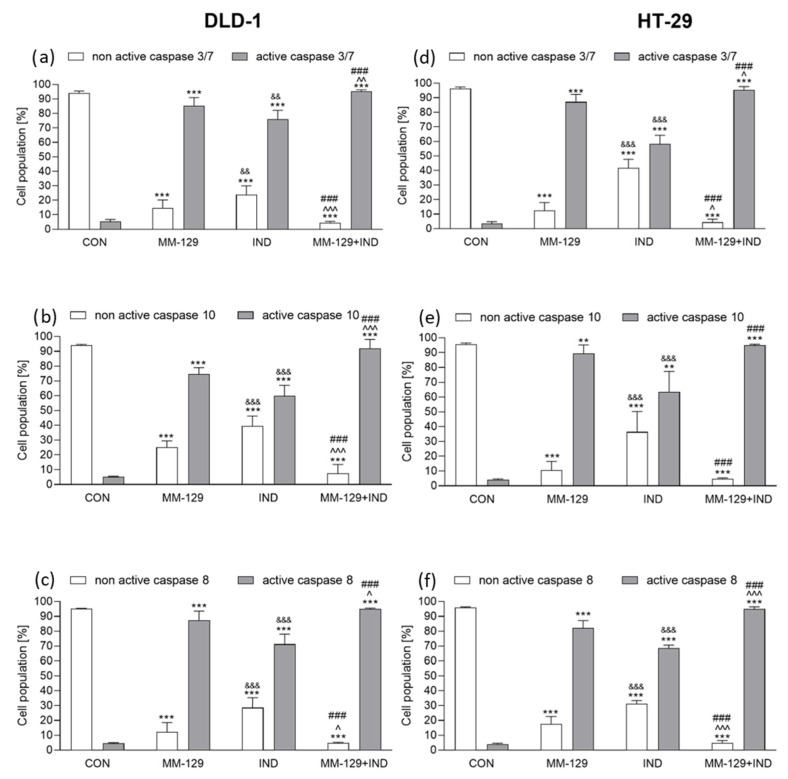
Flow cytometric analysis of caspase-3/7, caspase-10, and caspase-8 activation in DLD-1 (**a**–**c**) and HT-29 (**d**–**f**) colon cancer cells exposed to MM-129 (10 µM), IND (200 µM), and MM-129 + IND for 24 h. Values from three independent experiments performed in duplicate were presented as mean ± standard deviation (SD) and analyzed using one-way analysis of variance (ANOVA). ** *p* < 0.01, *** *p* < 0.001 vs. CON; ^ *p* < 0.05, ^^ *p* < 0.01, ^^^ *p* < 0.001vs. MM-129; ### *p* < 0.001 vs. IND; && *p* < 0.01, &&& *p* < 0.001 IND vs. MM-129.

Caspases are key regulators of programmed cell death, and there are two ways to induce this process: intrinsic and external. Initiator caspase-8 and -10 are part of the extrinsic pathway, which is activated via death receptors. This is after ligand binding death receptors accumulate in the cell membrane, which leads to the recruitment of adapter proteins. Subsequently, these proteins interact with procaspase-8 and procaspase-10 via the death effector domain, which in turn results in an activation of caspase-8 and caspase-10. In our study, we observed that in vitro, in both DLD-1 and HT-29, MM-129 activated caspase-8 and -10 stronger than IND applied alone. Then, 24 h incubation with both tested compounds resulted in similar level of caspase-8 activation in both cell lines and amounted to about 95% for DLD-1 (95.03% ± 0.4) and HT-29 (95.97% ± 1.4) cells. A similar trend occurred in the case of caspase-10 activation under the combined molecules. After 24 h incubation with MM-129 + IND, the rate of DLD-1 cells with an active caspase-10 was 92.13% ± 4.3, while in HT-29 cells, the value remained at 95.07% ± 0.7 (Figure 5b,c,e,f).

To fully describe the caspases’ activation in the latter stage of our research, we verified whether the tested compounds activated caspase-3/7. In contrast to the described above caspases-8 and -10, this is an effector caspase, which means that it affects the breakdown of cellular proteins and leads to complete cell death. It is also directly activated by the initiator caspases through the cleavage. With the use of flow cytometry, we checked if our compound combination was potent enough to activate a full caspase cascade. The obtained results revealed that MM-129 was generally more potent to activate caspase 3/7 in both tested colon cancer cell lines than IND alone. However, as in the previously observed changes in initiator caspase activation, co-administration of both molecules resulted in stronger caspase 3/7 activation in both DLD-1 and HT-29 cell lines. After 24 h exposure to both compounds, we detected an active form of caspase 3/7 in 95.26% ± 1.2 of DLD-1 and 95.46% ± 3.1 HT-29, which was significantly higher when compared to the control group (Figure 5a,d). 

### 3.6. AKT Signaling Pathway Was Impaired under the Impact of Compound Combination

**Figure 6:**
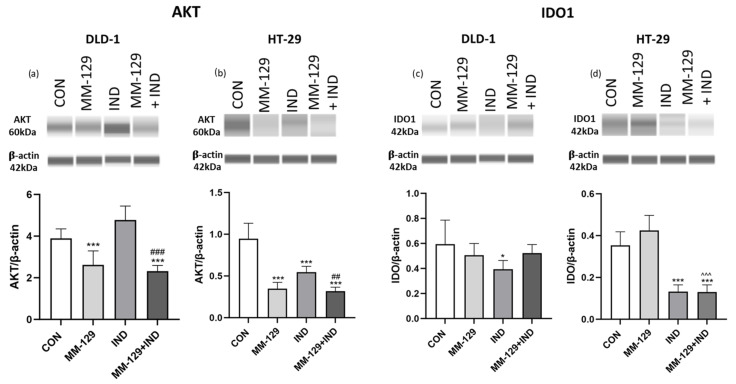
The downregulation of protein kinase B (AKT) (**a**,**b**) and indoleamine 2,3-dioxygenase-1 (IDO1) (**c**,**d**) exerted by MM-129 (10 µM), IND (200 µM), or MM-129 + IND toward DLD-1 and HT-29 colorectal cancer cells after 24 h exposition. The values were obtained from three independent experiments performed in duplicate. * *p* < 0.05, *** *p* < 0.001 vs. CON; ^^^ *p* < 0.01 vs. MM-129; ## *p* < 0.01, ### *p* < 0.001 vs. IND. Images and quantification were obtained by capillary protein separation and immunodetection. The uncropped blots are shown in the supplemental materials.

The abovementioned results indicated an antiproliferative and proapoptotic potential of the proposed compound combination. That prompted us to determine which of the signaling pathways was involved in causing such effects. It is well known that the PI3K/AKT pathway is highly involved in several cellular processes, necessary for cell survival. Among them, metabolism, proliferation, and apoptosis should be listed. Many of the anticancer drugs target this pathway. Due to this fact, we decided to test whether the proposed compound combination affects the expression of AKT protein kinase. What is interesting is that the obtained results demonstrated a strong inhibition of the protein’s expression in both cell lines under the simultaneous administration of MM-129 + IND. While we observed a favorable effect of MM-129 in both tested cell lines, IND used alone was insufficient to inhibit the AKT expression in DLD-1 cells, but it caused its downregulation in HT-29. Despite this, co-administration of the compounds resulted in AKT inhibition at a level comparable to the effect caused by the 1,2,4-triazine derivative. This suggests that the inhibition of AKT expression exerted by MM-129 was not impaired under the influence of IND (Figure 6).

### 3.7. Indoximod but Not MM-129 Downregulated IDO1 Expression

Indoximod was developed as an inhibitor of the kynurenine pathway. However, available studies focus mostly on the effects exerted by IND on the immune cells’ functions. The number of studies evaluating its direct effect on cancer cells is still limited. Additionally, the effect of MM-129 on the TRP metabolic pathway has not yet been studied. Taking these gaps into consideration, we included in our study an experiment that let us investigate whether the proposed compound combination is potent enough to inhibit the expression of the limiting enzyme of the TRP metabolite pathway. For this purpose, we checked whether the expression of IDO1 protein in DLD-1 and HT-29 colorectal cancer lines decreased under the impact of the tested compound combination. The presented results indicate that only IND was responsible for IDO1 inhibition. Interestingly, HT-29 cells turned out to be more sensitive to the proposed compound combination than DLD-1. In the former, exposure to both the KP inhibitor and its combination with MM-129 resulted in strong inhibition of the IDO1 enzyme. In DLD-1 cells, only the use of IND alone resulted in a weak decrease in IDO expression, and the combination of compounds did not exert a superior effect (Figure 6). 

## 4. Discussion

In our previous studies, we showed that MM-129 exerts an antitumor effect toward colon cancer zebrafish and mouse xenografts [15]. Due to the increasing interest of scientists in the role of immunological processes in cancer development, we extended our current research and checked whether the combination of 1,2,4-triazine derivative MM-129 with a kynurenine pathway inhibitor—indoximod (IND)—would result in a stronger anticancer response. Previous reports on the effects of IND focused on its immunological effects. However, it is not clear whether IND has a direct effect on the processes ongoing in colorectal cancer cells. In this study, we have presented, for the first time, that this compound combination exerted a stronger antitumor effect in the zebrafish model of colorectal cancer, which was particularly noticeable in DLD-1 xenografts. Moreover, we have shown that in vitro combination of compounds enhances apoptosis through decreasing mitochondrial potential, enhanced phosphatidylserine externalization, and caspase activation. Finally, we detected that only IND is responsible for the IDO1 inhibition. 

Zebrafish (*Danio rerio*) is used in oncological research thanks to its unique features. Among them is its transparent body, which allows for observations of phenomena at the single-cell level, ease of obtaining a large number of offspring, the possibility to obtain reliable results in a short time, and high gene homology to humans (approximately 70% of genes, including non-coding region), with these underlined as the most valuable [21,22,23]. However, this is not a model free from limitations. The temperature for conducting experiments using zebrafish is lower than the human body temperature, which may affect the observed processes. Also, drug administration through direct absorption from the zebrafish medium may impact the precision in compound dosing [24]. The model also poses technical difficulties: due to its small size, manipulation of the equipment and implantation of cells is highly challenging. In the presented study, simultaneous incubation of zebrafish colon cancer xenografts with MM-129 and IND resulted in a significant reduction of tumor cells manifested as a strong reduction of a human *GAPDH* gene expression. We assessed it by the qRT-PCR method due to its higher sensitivity [19], and further supported it by imaging. So far, it has been established that in vivo IND empowers T-cell activity toward eliminating cancer cells. It is manifested as inhibition of immunosuppressive Treg proliferation, enhanced differentiation of suppressor Tregs into anti-cancer Th17, and activation of effector T cells [13,25]. IND as a KP inhibitor reverses the effects of TRP depletion in the tumor microenvironment, which results in the reactivation of MAP4K3 and subsequent activation of mTORC1 activity, together with the inactivation of GCN2 kinase in T helper cells [26]. Those events are linked with favorable antitumor response [27,28,29,30]. Zebrafish as a model organism is relevant for immunological studies due to the presence of T cells, which are potent to differentiate into two subpopulations: antitumor Th1-like and protumor Th2-like [31,32]. This reflects processes similar to those ongoing in humans. Also, its immune system contains other immune cells, such as macrophages, natural killer (NK) cells, and neutrophils, which expand the scope of the use of zebrafish in immunooncological research [33,34,35,36]. Equally important, in the context of investigating KP’s role in cancer development, is the fact that TRP metabolizing enzymes were previously detected in zebrafish, and their involvement in various disorders was already studied using this model [37,38,39,40]. In the given study, we confirmed that the combination of tested molecules, meaning simultaneous targeting of cancer cells and KP enzyme, exerts a stronger antitumor response toward colon cancer xenografts than any of the used compounds alone. This is in line with reports, which reveal an enhanced tumor regression under the impact of KP inhibitors combined with chemotherapeutic agents [41,42,43]. Favorable results give a base to plan further experiments, focusing on exact immunologically driven processes responsible for an antitumor effect. This aspect will be the subject of our future study.

MM-129, meaning a 1,2,4-triazine derivative, is similar to roscovitine in terms of structure [15]. To the best of our knowledge, there are no reports that would describe 1,2,4-triazine derivatives or roscovitine as factors that influence IDO1 expression or any other KP elements. To verify if MM-129 influences the impact of IND toward IDO1, we assessed an expression of this protein in DLD-1 and HT-29 cells after 24 h with both tested molecules. What is highly important is that our results have shown that only IND downregulates IDO1 expression in both tested colon cancer cell lines. Due to the lack of the impact of MM-129 on the KP enzyme, we concluded that this molecule inhibited cancer growth only by disrupting processes ongoing directly in the malignant cells, whereas IND was additionally responsible for inhibiting the expression of the kynurenine pathway enzyme. 

While investigating in detail the IDO1 role in colon cancer development, we found a report that suggests that the enzyme is directly involved in colorectal cancer (CRC) proliferation, and that its inhibition improves colon cancer management [44]. Based on this, together with the abovementioned observation of IDO1 downregulation, we conducted experiments that verified whether the tested molecule combination exerts a favorable antiproliferative effect. For this reason, we conducted a test with the use of zebrafish embryos. It is a simple, reliable, and highly efficient tool for cancer drug screening and it was pointed as an assay complementary to commonly used MTT [45]. In our experiment, we showed that both compounds disturbed cell proliferation of zebrafish embryos; however, MM-129 exerted an antiproliferative effect on the earlier stage of cell division than IND used alone. The exposition on the compound combination resulted in an inhibition of cell proliferation at the same point of cell division as detected after the exposition on MM-129 only. This suggests that the antiproliferative effect observed in both groups exposed to tested molecules alone was not diminished by their co-administration. To verify these observations, we conducted a classical MTT assay and checked the cytotoxic effect exerted after 24 h of DLD-1 and HT-29 incubation with the tested compounds. At this stage, similarly to results obtained in zebrafish embryos, we showed an antiproliferative effect exerted by both compounds. Results obtained in the MTT test coincided with the outcome of the previous experiment and indicate an antiproliferative effect of both compounds when used alone, with MM-129 exerting a stronger reduction of cell viability. Again, the addition of IND to 1,2,4-triazine derivative did not reverse its effects. Reduced proliferation of cancer cells after IDO1 inhibition was previously reported by Hill et al., which supports our findings of the antiproliferative potential of IND [46]. We showed a lack of an overriding cytotoxic effect of the compound combination, which stays in line with the conclusion reported by Maletziki et al. In their research, they highlighted that even preconditioning of CRC cells with IND neither increases their chemosensitivity nor increases the cytotoxicity of 5-fluorouracil (5-FU), gemcitabine, and irinotecan [47]. Interestingly, in breast cancer cells, NLG-919, an indoximod prodrug, complexed with cyclodextrin, enhanced the cytotoxic effect of paclitaxel toward HeLa, and the authors observed it as a dose-depended effect [48]. Looking for discrepancies between their results and our observations, one can point to different levels of IDO1 expression between breast and colorectal cells. The abovementioned Maletziki et al. study reported that the level of IDO1 expression inversely affects cell sensitivity to indoximod, and higher expression of IDO1 in colorectal cancer cells than in breast cancer cells was previously observed [47,49]. This suggests also that the sensitivity of malignant cells to treatment with IDO1 inhibitors varies between tumor types and points to the complex role of the kynurenine pathway in cancerogenesis.

The obtained results led us to investigate molecular processes that could be affected by the simultaneous administration of MM-129 and IND. By the Western blot test, we assessed protein expression and noticed that in DLD-1 and HT-29 colon cancer cells, expression of AKT kinase decreased after 24 h exposure to the combination of molecules. It is well known that AKT is highly involved in cell proliferation, and it serves as one of the potential targets in colon cancer therapy [50]. In our previous studies, we showed that MM-129 was potent to decrease the expression of AKT in CRC cells. However, currently, there are no data on whether indoximod exerts any direct effect on this protein expression in colon cancer cells. The results obtained in the given study differed between cell lines and indicated that IND alone was potent to inhibit AKT expression in HT-29 but did not exert this effect in DLD-1. It is worth noticing that Bishnupri et al. reported a link between KP, IDO1 inhibitors, and dysregulation of the AKT signaling pathway in colorectal cancer cells. They noticed that KP metabolites increased the activation of AKT in CRC cancers, and they suggested that IDO1 inhibition synergized with the cytotoxic chemotherapeutics targeting the AKT pathway [5]. In addition, Santhanam et al. reported an increased CRC proliferation in the AKT-dependent manner under the impact of kynurenine—the first metabolite of TRP in the kynurenine pathway. This effect was reversed after the exposition of an IDO1 inhibitor, INCB24360 [51]. 

Finally, we checked whether the proposed compound combination exerted a proapoptotic effect. For that purpose, we observed changes in mitochondrial membrane potential; externalization of phosphatidylserine; and activation of caspases, i.e., known markers of cell death. The obtained results allowed us to conclude that the simultaneous use of both compounds impacted the induction of apoptosis in colon cancer cells. The available data also show that human tumor cells are more sensitive to gemcitabine with simultaneous IDO1 downregulation [52]. Interestingly, a combination of gemcitabine with curcumin evoked more intense apoptosis in cancer cells [47]. The latter was previously identified as a kynurenine pathway inhibitor in cancer cells [53]. In this given study, we also showed that the proapoptotic effect of molecule combination was additionally exerted via an intense caspase activation. Available reports demonstrate that epacadostat, as well as 1-L-MT, another IDO1 inhibitor, led to the activation of a caspase in cancer cells [54,55]. A similar result to this one was obtained in HeLa cells, in which simultaneous use of IND with doxorubicin resulted in a significant increase in caspase-3 activity with concomitant apoptosis of these cells [56].

In the presented study, we validated that simultaneous targeting cancer cell along with downregulation of IDO1 expression results in strong antitumor effects in zebrafish xenografts. Also, both tested compounds showed proapoptotic effects to varying extents, whereas only IND inhibited IDO1 expression. This extension of action might be a reason for the favorable results observed in Danio rerio. Since the anticancer effect of IDO1 inhibition requires the presence of immunological mechanisms, a clear effect of potentiating the anticancer effect of the combination of compounds is difficult to demonstrate in vitro. However, in a complex organism, the effect of the KP enzyme inhibitor is revealed, intensifying the anticancer effect exerted by MM-129. Moreover, the presented observations indicate the proapoptotic effect of IND on colorectal cancer cells, which expands the current knowledge about its mechanism of action.

In the end, we would like to highlight the difference in response between DLD-1 and HT-29 cells to the proapoptotic effects of the tested compounds. DLD-1 cells turned out to respond better to the proapoptotic effects of both the combination of compounds and MM-129 or IND administrated alone. This was particularly visible in the reduction of mitochondrial potential. In this experiment, both compounds administered separately decreased mitochondrial potential to a similar extent. We also observed a stronger reduction of human *GAPDH* mRNA in DLD-1 zebrafish xenografts under the combined administration of compounds than in HT-29 xenografts. As mentioned previously, DLD-1 cells are histologically the most similar to a primary tumor, whereas the HT-29 line lets us assess whether multidrug resistance, absorption of nutrients, and chemically induced differentiation of enterocytes occur. Stronger sensitivity to apoptosis of DLD-1 when compared to HT-29 cells was reported in previous reports, which stays in line with the presented results [57,58,59,60]. Various mechanisms, such as a higher expression of glucose regulated protein 78 in DLD-1 or a stronger expression of mitochondrial cyclooxygenase-2 (COX-2) in HT-29, were reported as a reason of this discrepancy [58,60]. What is highly interesting is that Cesario et al. conducted a study that revealed an interplay between IDO1 and COX-2 in cancer [61]. In our study, we noted a stronger downregulation of IDO1 expression in HT-29 cells, one that might shape our future work on existing crosstalk. On the other hand, in the zebrafish DLD-1 xenografts exposed to MM-129 or IND alone, we observed an inhibition of tumor development at a similar level, but in HT-29 xenografts, IND alone inhibited tumor development more strongly than MM-129 alone compared to the control group. We also observed different response of DLD-1 and HT-29 cells on IDO1 inhibition by IND. Confronting these observations, the question arises as to whether there is a relationship between the sensitivity of cells to apoptosis and the role of elements of the KP pathway in the development of colorectal cancer. These intriguing results require in-depth research and will become the subject of our further work focusing on the role of KP-dependent immune processes involved in the development of colorectal cancer.

When considering the future usefulness of the presented results, the question arises about the possibility of their practical application. By discussing this issue from a broader perspective, we would like to refer to reports regarding the development of new compounds potentially useful in CRC therapy. One of the biggest challenges in oncology is targeted therapy. For this purpose, new forms of drugs are created and successfully tested, including liposomes, nanoparticles, and cubosomes [62,63,64,65]. Their use allows for the achievement of preferential accumulation in nonspecific cancer cells, as well as a reduction of toxicity in other vital organs. MM-129, as a new molecule, has not yet been tested for possible formulations into modern forms of the drug. On the other hand, IND and its prodrug have already been subjected to such tests [66]. What is particularly important is that nanoparticles and liposomes that were created contained IND together with compounds classified as classical chemotherapy [67,68]. Their use was associated with greater effectiveness in experimental models [12]. This gives rise to the hypothesis that the tested compound combination will have a chance of exerting anticancer effects in a moder formulation. Following the approach of targeted therapy and maximizing the effectiveness and safety of therapy, we plan to conduct research that will bring us closer to developing this form of combination of compounds. We hope that this will increase its clinical potential.

## 5. Conclusions

To sum up, we showed for the first time that the combination of the 1,2,4-triazine derivative MM-129 with the inhibitor of the kynurenine pathway, indoximod, results in a stronger antitumor response toward colon cancer cells. This indicates that simultaneous targeting of processes ongoing in the tumor cell, together with the inhibition of the kynurenine pathway enzyme, is a right and promising approach in designing future therapeutic options in the fight against colorectal cancer. Undoubtedly, the presented studies need further investigation, especially with the deep focus on immunologically driven changes responsible for an antitumor response. 

## Data Availability

The data presented in this study are available in this article (and supplementary material).

## Supplementary Materials

The following supporting information can be downloaded at: https://www.mdpi.com/article/10.3390/cancers16010122/s1, Full pictures of the Western blots.
