# Supplementary material for: Assessment of an Anticancer Effect of the Simultaneous Administration of MM-129 and Indoximod in the Colorectal Cancer Model
Source: Cancers (Basel). 2023 Dec 26;16(1):122. doi: 10.3390/cancers16010122 (PMC10778160; doi:10.3390/cancers16010122)
Supplement: Supplementary file 1 [file cancers-16-00122-s001.zip › WB original blot.pdf]

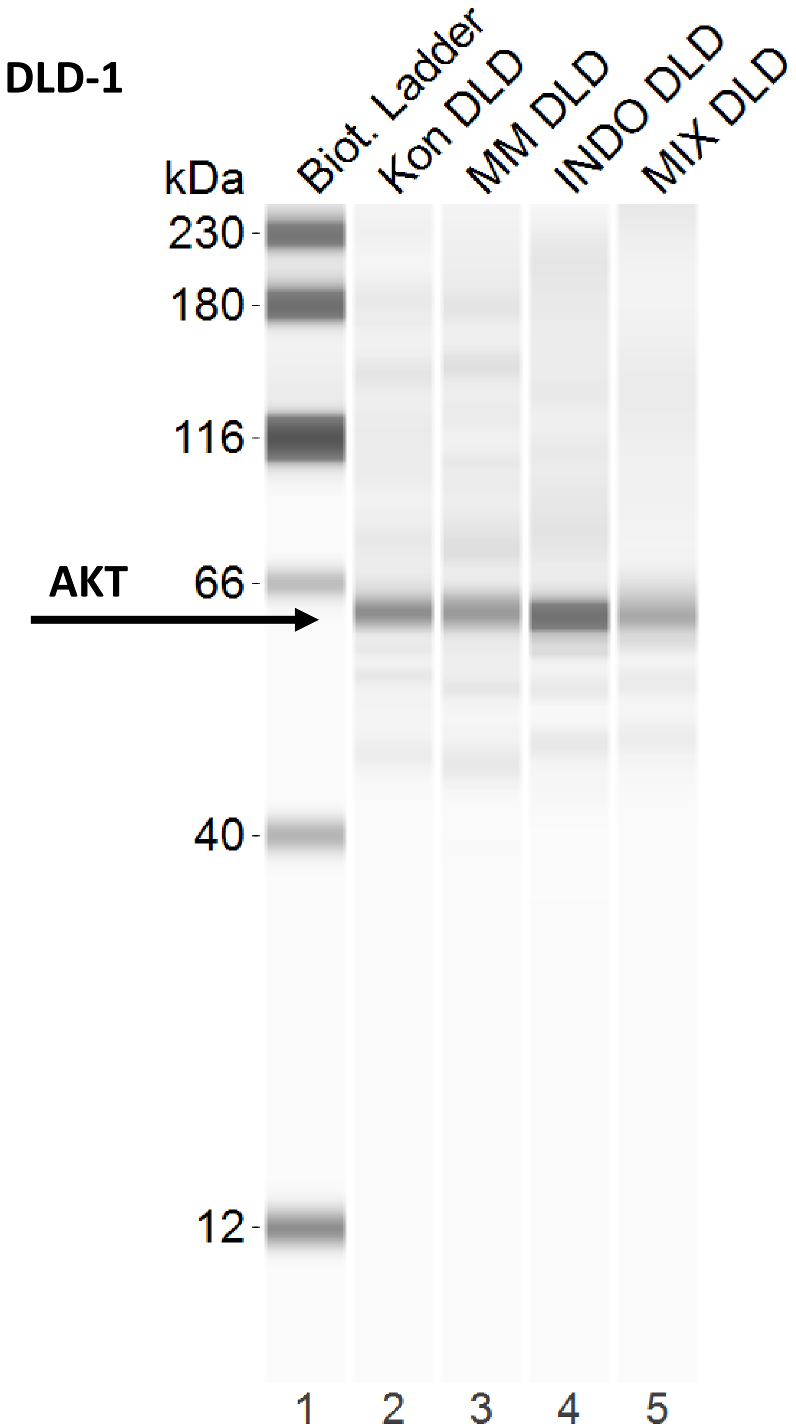

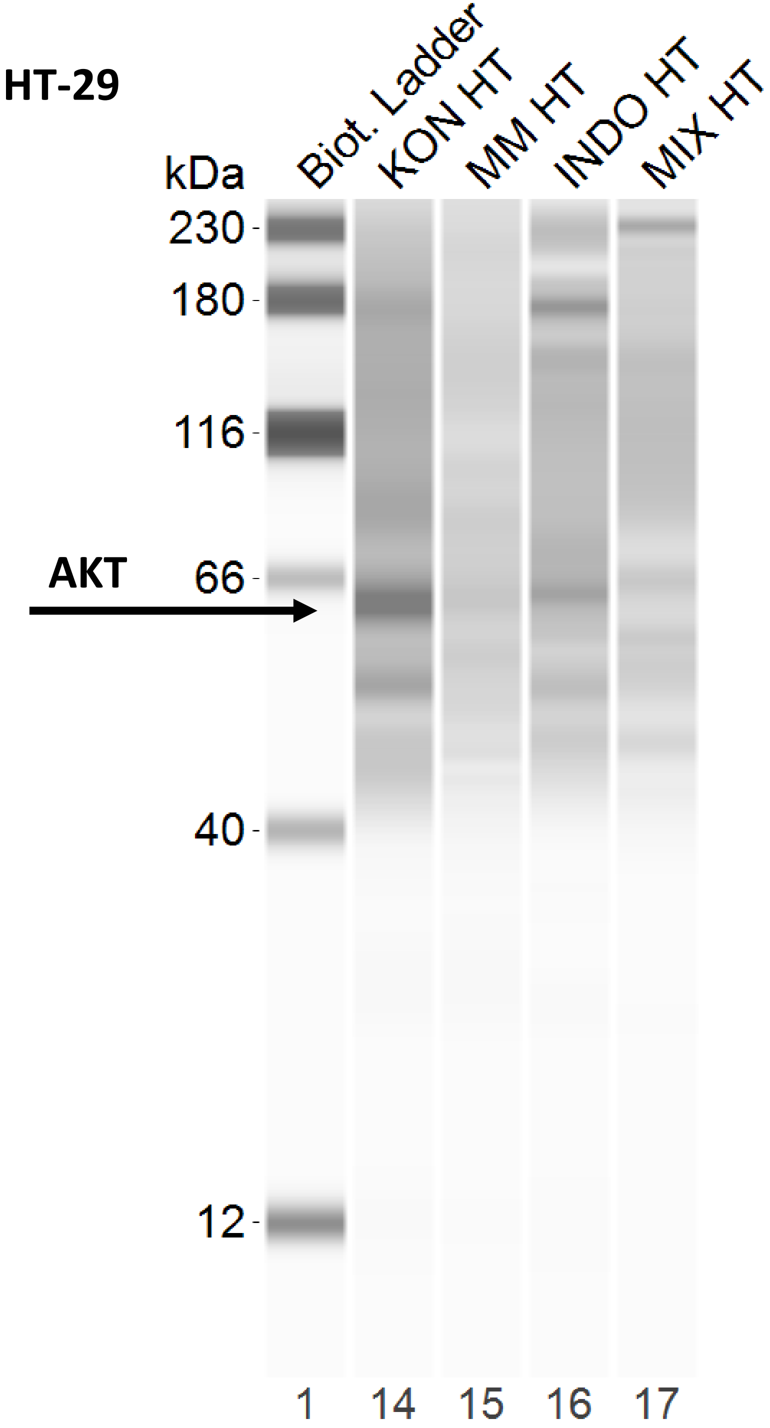

AKT expression:

Order of loading:
Ladder
CON
MM-129
IND
MM-129+IND

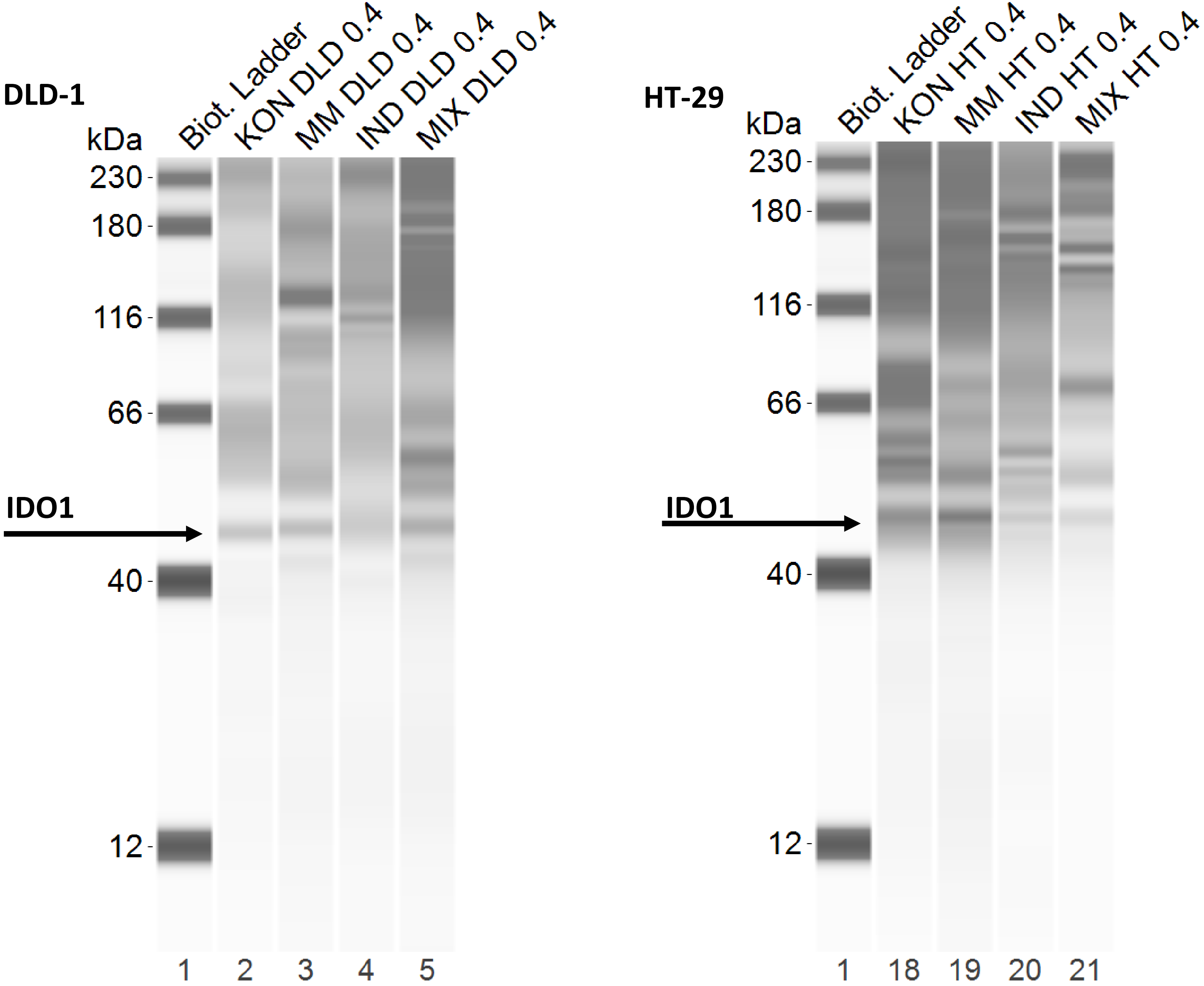

IDO1 expression:

Order of loading
Ladder
CON
MM-129
IND
MM-129+IND

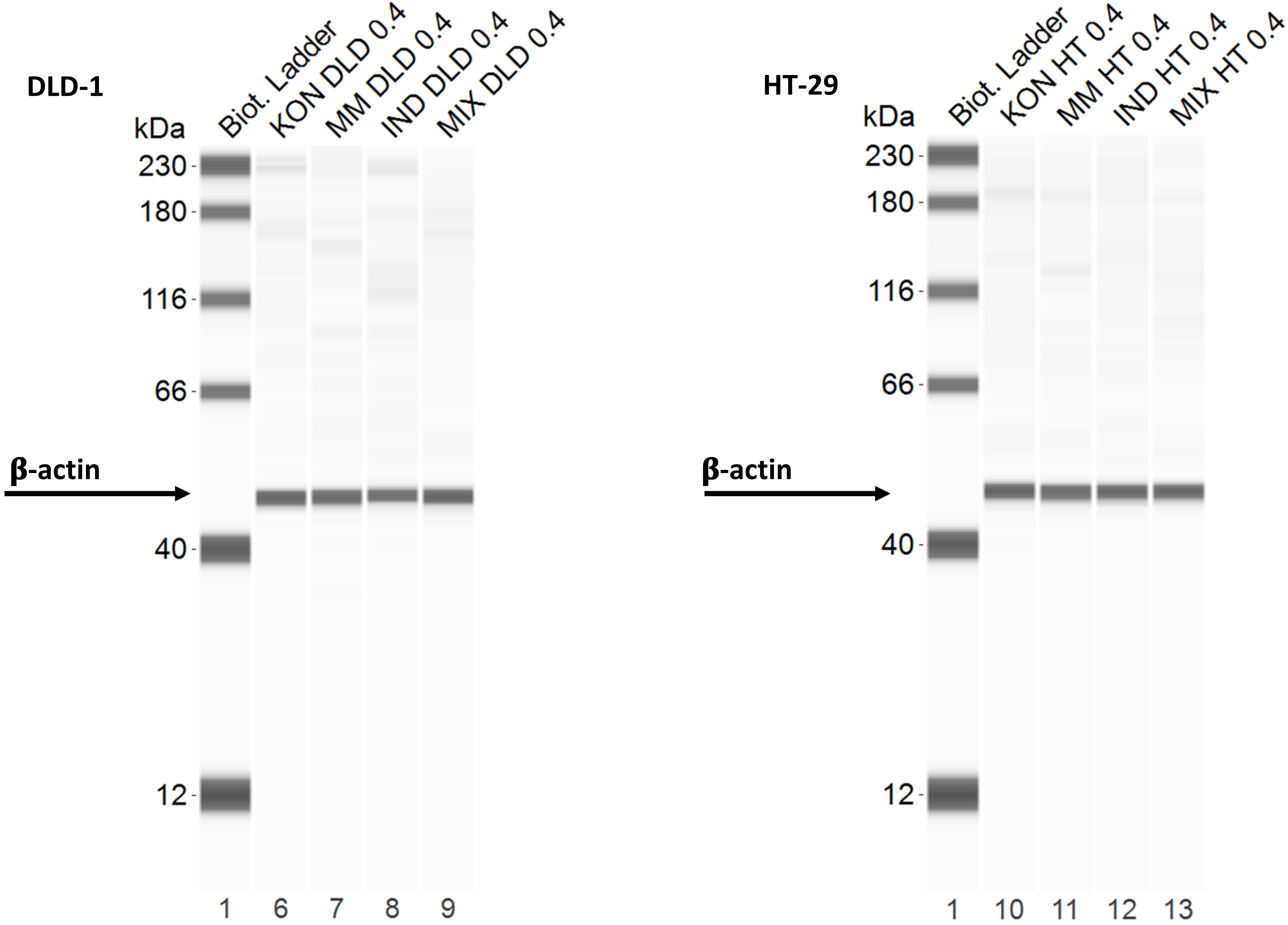

β-actin expression:

Order of loading
CON
MM-129
IND
MM-129+IND
